# An Improved Two-Shot Tracking Algorithm for Dynamics Analysis of Natural Killer Cells in Tumor Contexts

**DOI:** 10.3390/bioengineering11060540

**Published:** 2024-05-24

**Authors:** Yanqing Zhou, Yiwen Tang, Zhibing Li

**Affiliations:** 1 School of Physics, Sun Yat-sen University, Guangzhou 510275, China; zhouyq86@mail2.sysu.edu.cn; 2 Department of Physics, Northeastern University, Boston, MA 02115, USA; tang.yiwe@northeastern.edu; 3 School of Science, Shenzhen Campus of Sun Yat-sen University, Shenzhen 518107, China

**Keywords:** natural killer cells, cell polarization, small-object tracking, machine learning, dynamics analysis

## Abstract

Natural killer cells (NKCs) are non-specific immune lymphocytes with diverse morphologies. Their broad killing effect on cancer cells has led to increased attention towards activating NKCs for anticancer immunotherapy. Consequently, understanding the motion characteristics of NKCs under different morphologies and modeling their collective dynamics under cancer cells has become crucial. However, tracking small NKCs in complex backgrounds poses significant challenges, and conventional industrial tracking algorithms often perform poorly on NKC tracking datasets. There remains a scarcity of research on NKC dynamics. In this paper, we utilize deep learning techniques to analyze the morphology of NKCs and their key points. After analyzing the shortcomings of common industrial multi-object tracking algorithms like DeepSORT in tracking natural killer cells, we propose Distance Cascade Matching and the Re-Search method to improve upon existing algorithms, yielding promising results. Through processing and tracking over 5000 frames of images, encompassing approximately 300,000 cells, we preliminarily explore the impact of NKCs’ cell morphology, temperature, and cancer cell environment on NKCs’ motion, along with conducting basic modeling. The main conclusions of this study are as follows: polarized cells are more likely to move along their polarization direction and exhibit stronger activity, and the maintenance of polarization makes them more likely to approach cancer cells; under equilibrium, NK cells display a Boltzmann distribution on the cancer cell surface.

## 1. Introduction

**Figure 1:**
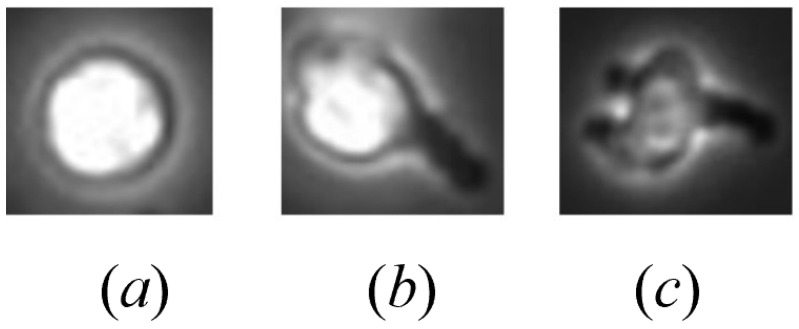
Examples of different morphologies of natural killer cells: (**a**) A non-polarized natural killer cell (NP-NKC), with its intracellular substances roughly evenly distributed, presents a regular spherical shape overall. (**b**) A simple polarized natural killer cell (P-NKC), characterized by its irregular shape, with a visible cell body and a slender black “tail”. This is due to the reorganization of migratory substances within the cell, causing symmetry to be disrupted, a phenomenon referred to as polarization, with the tail pointing towards the cell body as the polarization direction. (**c**) Complex polarized cells differ from simple polarized cells in that they have multiple tails and multiple polarization directions.

Natural killer cells (NKCs) are a crucial part of the innate immune system. Despite their lower killing activity compared to lymphocytes like T cells, NKCs can non-specifically eliminate tumor and virus-infected cells without the need for prior sensitization [1]. Moreover, NKCs possess the ability to undergo morphological changes upon stimulation [2]. As illustrated in Figure 1, NKCs of different forms exhibit distinct motility traits. Understanding the morphology of cells and the influence of the cancer cell environment on the motility of NKCs is becoming increasingly important in academic research [3,4]. By activating NKCs to change their morphology, they can be more effectively utilized for immune responses against cancer cells [5,6]. Therefore, quantitative analysis of NKCs’ immunological data to understand their group dynamics is necessary, yet this research is challenging, and related work remains limited [7,8,9,10]. Accurate detection, classification, and stable tracking of NKCs’ coordinates are imperative for such analysis. The two-shot tracking algorithm, widely used in multi-object tracking (MOT) tasks, offers a promising solution [11]. This approach divides the MOT process into two independent parts: 1. A detector for analyzing and identifying objects in single-frame images; 2. A data-association component that links detected objects across frames, assigning IDs to maintain continuous trajectories. Because these two parts operate independently, the detector is interchangeable, allowing users to select and modify the detector according to their needs. With the development of deep learning, particularly advancements in image detection processes, we are better equipped to analyze the morphology of NKCs and extract key points for analysis [12,13,14,15]. This has led to increased accuracy and stability in multi-object tracking algorithms. For example, tracking methods like DeepSORT and ByteTrack have been widely applied in industry, demonstrating excellent performance in pedestrian and vehicle tracking. However, tracking small objects such as NKCs in complex backgrounds still poses many challenges [16,17,18,19].

Firstly, the performance of tracking algorithms highly depends on the accuracy of detectors, leading to the following issues: (1) High-accuracy detectors often require extensive datasets for training, but, in many experiments, our original datasets are limited; (2) Building the original training set often consumes a lot of time and effort, and the generalization ability of deep learning is limited. Experimental conditions or imaging equipment changes often reveal the variability in datasets, making it difficult to accurately detect with existing weights. This means that we need to add corresponding training data for weight adjustments, increasing time costs; (3) The small image area occupied by NKCs makes it difficult to judge cells based on single-frame information in complex backgrounds, especially when cells are occluded or interfered with, relying instead on context information for judgment.

Secondly, the cell matching issue arises when detecting cells. For small targets like NKCs, matching presents the following difficulties [19]: (1) Due to the few pixels occupied by cells in images, their features are few and unstable; (2) In dense scenes, cells easily overlap and occlude each other, making it challenging for tracking algorithms to maintain target identity information; (3) In time-lapse photography, the relative speed of P-NKCs is often significant, meaning that the displacement of the cell itself over two frames relative to the length of the target is much larger, complicating the capture of trajectories by algorithms.

Due to these issues, while common industrial algorithms such as DeepSORT and ByteTrack are capable of tracking conventional targets like pedestrians, they encounter several challenges when tracking natural killer cells (NKCs) in complex environments. Therefore, the aim of this paper is to optimize traditional algorithms for the precise tracking of small targets like NKCs in complex environments. At the same time, we expect to use this new tracking algorithm to simultaneously complete the morphological analysis and dynamic tracking of NKCs. By integrating these advancements, we provide a comprehensive examination of NKCs’ behavior, with a particular focus on how their polarized states, environmental temperature, and proximity to cancer cells influence their motility. This dual approach, combining advanced image processing with a meticulous analysis of NKCs’ group dynamics, offers a novel pathway for understanding their role in immune responses and presents potential strategies for enhancing their effectiveness in cancer immunotherapy.

## 2. Materials and Detection Process

### 2.1. Experimental Materials

**Figure 2:**
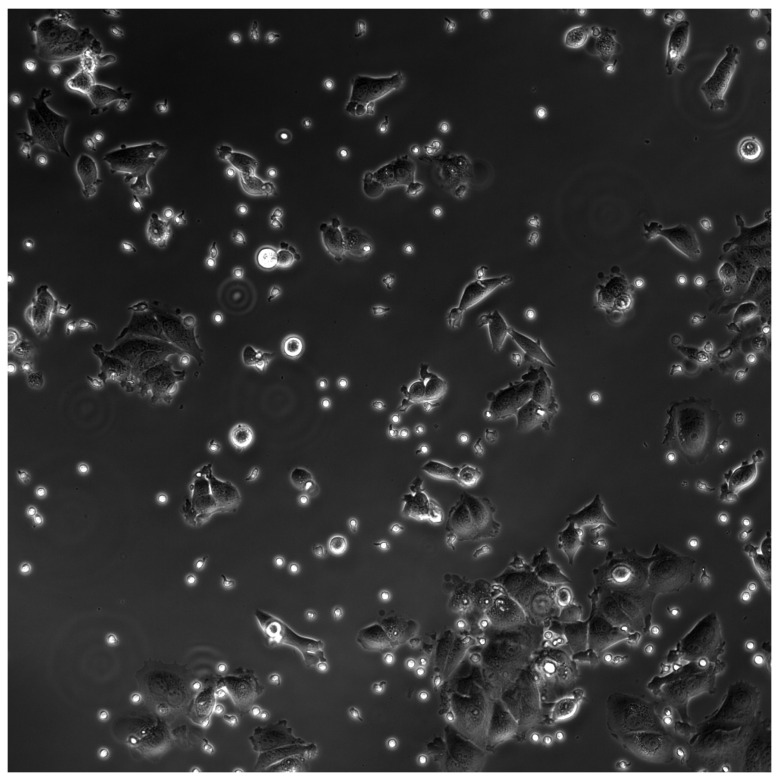
Example of an image depicting natural killer cells in the cancer cell environment. The small fluorescent cells represent natural killer cells. In the experiment, the ratio of polarized to unpolarized natural killer cells is approximately 1:1 (52:48). The irregularly shaped and larger cells in the images are cancer cells, which do not exhibit movement during the experiment and only show minimal morphological changes. The only difference observed in the experimental group without cancer cells in the images compared to the images above is the absence of cancer cells within the field of view.

The experimental materials for this study were supplied by Professor Jue Shi from Hong Kong Baptist University. The natural killer cells utilized in this study were extracted from fresh human blood, provided by the Hong Kong Red Cross, and were sustained and activated outside the body using Interleukin-2 (IL-2). The cancer cells chosen for the study were U-2 OS, originating from bone cancer. Cell imaging was conducted using a Nikon TE2000-PFS inverted microscope sourced from Nikon Corporation, Tokyo, Japan, capturing images every 30 s with a motorized stage and a 20X objective (NA = 0.95). The microscopic images had a resolution of 2048 × 2048 pixels, each pixel representing 0.65 μm in reality. A series of experiments were conducted: 1. The migration of natural killer cells in an environment devoid of cancer cells was observed, measuring cell performance at 30 °C and 37 °C, with an average of about 80 cells per frame, and each experimental set observed 601 frames, repeated three times; 2. The movement of natural killer cells in a cancer cell environment was observed, where the cancer cells were fixed in the culture dish, and their positions remained almost unchanged, each frame containing about 220 cells, totaling 1080 frames, also repeated in three sets. The images from the second experiment can be seen in Figure 2. Throughout the experiments, no cell death occurred, and the natural killer cells maintained good motility. For detailed specifics of the experiments, please refer to Professor Jue Shi’s paper [20,21].

### 2.2. Detection Process

In the context of our experiments with natural killer cells (NKCs), both understanding their positional data and accurately classifying their morphologies are crucial for processing their motion data. This necessitates a prior step of cell detection within individual frames before moving on to cell tracking. With the advancement of deep learning, contemporary detection algorithms, such as ResNet [22], Faster R-CNN [12], SSD [23], Transform [24], GCN [25], and YOLO [26], have significantly advanced, offering robust solutions for complex image analyses. Among these, we have opted for the YOLOv5 framework for cell detection, renowned for its speed and efficiency. YOLOv5, with its potent capability for image understanding, not only facilitates the detection of cell morphologies but also allows for the identification of cell key points through the YOLO-POSE [27] extension.

Due to the large size of the original images (2048 × 2048 pixels) and the small size of the cell images (around 30 × 30 pixels), training the network with the original images did not yield good results. To address this, we processed the original images by cropping, adding random noise, and stitching to enhance the data. Images were extracted from different experimental datasets, and 150 images were selected, with 100 images depicting natural killer cells in a cancer cell environment and 50 images depicting natural killer cells in an environment without cancer cells. These images was expanded to 9000 images. The YOLOv5x network architecture was chosen, with a batch size of 64, and the model was trained for 300 epochs using stochastic gradient descent.

**Figure 3:**
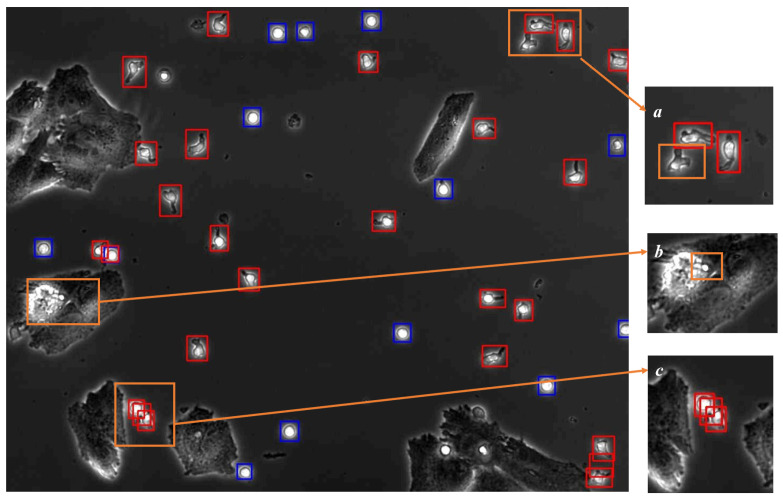
Example of our detection algorithm results, where the blue boxes mark NP-NKCs, and the red boxes are for P-NKCs. It shows that, although the overall detection accuracy is as high as 92%, there still exist the following types of errors: (**a**) Due to insufficient training samples, the algorithm’s generalization ability is limited, resulting in a small number of NKCs not being successfully detected; (**b**) The complexity of the background makes it difficult to detect NKCs near cancer cells using only the current frame; (**c**) Targets are redundantly boxed, a frequent occurrence in densely populated cell areas.

The detection accuracy reached 96% in a non-cancer cell environment and 92% in a cancer cell environment, having reached a relatively high level. However, as shown in Figure 3, the following two types of errors still occurred: Firstly, the omission of targets due to a limited training set affected the algorithm’s generalization capabilities and significant background interference hindered accurate target identification; Secondly, targets were over-detected in scenarios where NKCs appeared densely or overlapped, and when NKCs’ morphological traits were not distinctive, leading to their misclassification as both P-NKCs and NP-NKCs. Despite these errors being relatively infrequent, the sheer number of targets that need to be detected in each frame means that these mistakes can accumulate, leading to significant challenges for subsequent tracking efforts. This highlights the importance of refining our detection approach to better manage the complexities of tracking a large number of small targets within these images.

## 3. Improvements in Tracking Process

Once the detection process is completed, we need to perform data association to correlate the detection results across frames for target tracking. This involves associating the targets detected in consecutive frames and assigning them continuous IDs to achieve persistent tracking of the targets. The need for accurate and continuous tracking of targets has led to the evaluation of two widely used metrics for tracking algorithms: MOTA and IDF1. MOTA primarily considers the accuracy of the tracking algorithm, providing a comprehensive assessment of missed detections, false positives, and mismatches, focusing more on whether the targets are successfully “tracked”. In contrast, the IDF1 metric focuses on maintaining target continuity, meaning it concerns whether the tracked trajectories are continuous for a particular target. For these metrics, achieving a score of 1 signifies the optimal tracking performance of an algorithm, with higher scores reflecting better effectiveness in tracking.

### 3.1. Analysis of Tracking Algorithm Limitations for NKCs

Before we delve into the principles and analysis of multi-object tracking (MOT) algorithms, it is crucial to introduce two commonly used tracking metrics: MOTA [28] and IDF1 [29]. These metrics evaluate the performance of an algorithm from two distinct perspectives. MOTA is an indicator that integrates the algorithm’s performance in terms of missed detections, false positives, and identity switches, offering a measure of whether targets are accurately represented in their trajectories by accounting for misses and false alarms. In contrast, the IDF1 metric focuses on maintaining target continuity and accurately tracking the movement of targets, paying closer attention to the consistency and stability of the tracking identities. For these metrics, achieving a score of 1 signifies the optimal tracking performance of an algorithm, with higher scores reflecting better effectiveness in tracking.

We integrate these two MOT tracking metrics with the target-tracking trajectory plots to assess the tracking performance of DeepSORT [30] and ByteTrack [31], two commonly employed algorithms in industrial settings, on NKCs. Additionally, we analyze the performance of the tracking algorithms by comparing their performance on the standard target dataset MOT17 and examining their underlying principles.

#### 3.1.1. DeepSORT

The DeepSORT algorithm, developed by Wojke et al. [30], is a multi-object tracking algorithm that has demonstrated excellent performance on many common tracking datasets, such as MOT15 and MOT17, thus finding widespread application in the industry. The innovation of this algorithm lies in the integration of a ReID (Person Re-identification) network and cascaded matching for multi-object tracking. ReID is a feature extraction algorithm based on convolutional neural networks, and, despite its name implying “Person Re-identification”, its scope extends beyond humans to encompass the extraction of appearance features from various objects. In DeepSORT, the ReID network is utilized to compress target images into feature vectors, and the similarity of appearance between targets is compared by calculating the cosine distance of vectors. Subsequently, DeepSORT matches the most visually similar targets between consecutive frames through cascaded matching. Cascaded matching involves optimizing the overall matching effect by comparing and matching targets layer by layer, thereby reducing the possibility of incorrect matches. For targets lost during tracking, DeepSORT retains their appearance features, but the probability of relocating these targets decreases with the increase in loss time. Therefore, for those lost targets, DeepSORT arranges their appearance matching with detection frames later, and the priority of matching gradually decreases with the increase in loss time until matching ceases.

**Figure 4:**
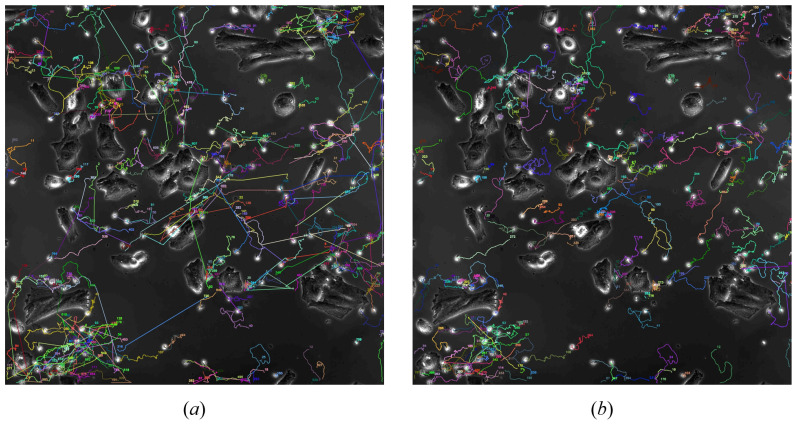
(**a**) The trajectory images obtained by the DeepSORT algorithm after tracking the same 100 frames show a large number of crossing lines in the trajectories. These errors are due to its adoption of appearance similarity matching. (**b**) The trajectory images obtained by the ByteTrack algorithm after tracking the same 100 frames do not exhibit the apparent matching errors seen with DeepSORT, thanks to its use of IoU matching. However, the tracks it produces are shorter, and the continuity of tracking is weaker. We provide a more detailed demonstration of ByteTrack’s tracking deficiencies in Figure 5.

We applied DeepSORT to our self-labeled dataset of NKCs in cancer cell environments, using manually labeled data as ground truth for metric calculation, and compared it with its performance on the conventional pedestrian dataset MOT17. The trajectory plots of target tracking are shown in Figure 4, and the results of detection metric calculations are presented in Table 1. From the trajectory tracking plots, we observed numerous erroneous trajectories, and the computed metrics indicated significantly poorer accuracy and continuity compared to its performance on the conventional pedestrian dataset MOT17. The subpar performance of DeepSORT in tracking NKCs can be attributed to two main reasons: 1. The limited cellular morphology information of NKCs makes it challenging to extract information, with the ReID network achieving only 79.2% accuracy after training; 2. The morphology of NKCs is not fixed, with the cell’s morphology constantly changing, making the approach of using appearance information for matching impractical.

#### 3.1.2. ByteTrack

In contrast to the appearance-based matching used by DeepSORT, ByteTrack focuses solely on the movement of targets. It employs Kalman filtering to predict the likely position of a target in the next frame (predicted box) and associates trajectories with the latest detected targets by calculating the Intersection over Union (IoU) values between predicted boxes and detection boxes. The innovation of ByteTrack lies in categorizing targets detected by the detection algorithm into two types: low-confidence targets and high-confidence targets. It considers high-confidence targets as definitely existing, while low-confidence targets might have low detection scores due to reasons such as occlusion. ByteTrack ingeniously uses low-confidence detection boxes, allowing the algorithm to better utilize information from the detection algorithm and effectively mitigate the tracking issues of partially occluded targets.

For ByteTrack, unlike DeepSORT, we observed from the target tracking trajectory plots that it did not exhibit a large number of unreasonable trajectories. Moreover, it outperformed the DeepSORT algorithm in various tracking metrics. However, ByteTrack still fell short of our expectations. Although its performance on the MOTA metric was similar to that on the MOT17 dataset, the IDF1 metric was significantly worse, indicating poorer stability and continuity in tracking despite the detection algorithm being relatively qualified. This situation can be attributed to the following three reasons: 1. As shown in Figure 6b, ByteTrack only utilizes the motion information of targets for prediction. Due to the small size of NKCs, even slight positional deviations can significantly affect the Intersection over the Union (IoU) value of the match, making it difficult to track NKCs with irregular or high-speed movements. Additionally, due to the short preservation time of target feature information, NKCs lost in the middle of tracking will be identified as new cells rather than being re-associated with past trajectories; 2. As illustrated in Figure 6d, in areas of target overlap, the detection algorithm produced multiple detections, with these erroneous bounding boxes being considered as trajectories; 3. As depicted in Figure 6g, ByteTrack relies on the low confidence of occluded targets for identification. However, in the case of small-target detection, often the confidence of the targets becomes close to zero when they are occluded or deformed. With limited training data, it becomes difficult to handle cells in complex backgrounds.

### 3.2. New Tracking Network

Through the analysis of the shortcomings of DeepSORT and ByteTrack, we propose the following three improvement strategies.

#### 3.2.1. Distance Cascade Matching

**Figure 5:**
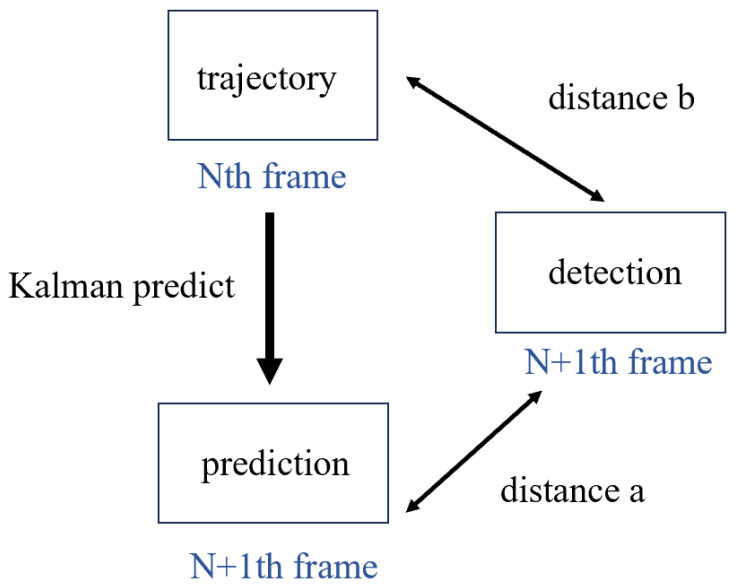
Two kinds of distance. In this figure, trajectory refers to the historical movement information of targets. Prediction refers to the inferred position of the target in frame N + 1, which is derived through Kalman filtering using the trajectory data—specifically, the known position and velocity from frame N. Detection refers to the actual position of targets in frame N + 1, as determined by the detection algorithm. At this point, we need to match the detection in frame N + 1 with the trajectory in frame N. Here, we propose distance “a”, which is the difference between the actual position and the predicted position, and distance “b”, which is the displacement of the actual position compared to the previous frame.

From the results discussed earlier, it is apparent that, in the process of linking frames for NKCs, motion information proves to be a more suitable criterion for matching than appearance data. However, due to the complexity of NKCs’ movement, Kalman filtering struggles to predict cell positions accurately, making the Intersection over Union (IoU) metric too stringent and less effective. Therefore, a more lenient matching criterion than IoU is required. We adopt a weighted distance calculation as the final matching criterion, as illustrated in Figure 5. Distance “a” represents the distance from the trajectory position predicted by Kalman filtering to the position of the detection box, whereas distance “b” is the distance between the target’s position in the previous frame and the current detection box. If a target’s movement is highly predictable, we can expect distance “a” to be smaller, making it a primary basis for matching. Conversely, distance “b” makes no assumptions about the target’s movement and is more suitable for matching when the movement lacks regularity. Our weighting method allocates a total weight of 1 to distances “a” and “b”, increasing the weight of distance “a” in proportion to the velocity of NKCs calculated by Kalman filtering. Finally, we obtain a distance matrix between detection boxes and trajectory boxes. We aim to find an optimal matching that minimizes the total distance among matched targets while ensuring that the matching adheres to physical constraints. Therefore, we first set a distance threshold as three times the cell’s side length, considering detection boxes beyond this threshold as unlikely to be matched. Subsequently, we employed the Hungarian algorithm to compute the optimal matching for the targets.

**Figure 6:**
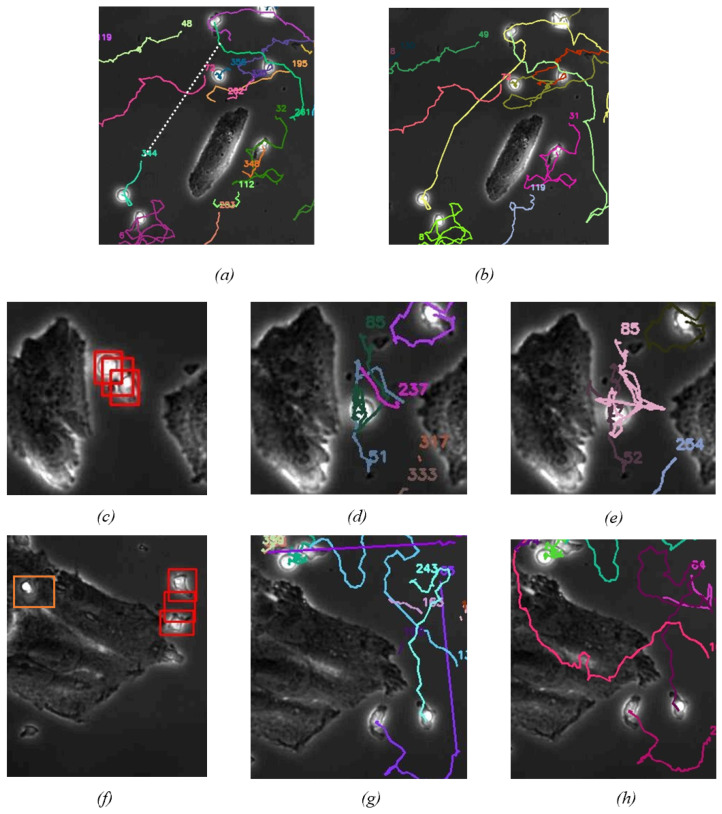
This image demonstrates how our algorithm has enhanced tracking performance: (**a**) ByteTrack loses track of fast-moving NKCs, missing parts of the trajectory lines, and identifies them as new targets upon re-tracking; (**b**) Our use of distance-based cascade matching effectively tracks these fast-moving NKCs; (**c**) The detection algorithm produces redundant detection boxes in densely populated cell areas; (**d**) Due to detection errors, ByteTrack generates redundant and broken trajectory lines; (**e**) After incorporating Overall Suppression, we eliminate incorrect trajectory lines, resulting in more stable tracking outcomes; (**f**) The detection algorithm fails to detect relevant cells against a background of cancer cells; (**g**) Due to missed detections by the detection algorithm, NKCs passing over the surface of cancer cells are not tracked; (**h**) After employing the Re-Search algorithm, we successfully achieve better tracking of NKCs in complex environments by searching for prominent areas near cells.

After employing motion information for matching, we opted for a cascaded matching algorithm in our tracking process. The purpose of this approach is to temporarily retain trajectories that have lost tracking, enabling them to be further tracked when the target reappears in the field of view. This method records the time *t* when a trajectory loses tracking and matches trajectories with the same loss time *t* to detection boxes on the same level. Trajectories with shorter loss times *t* are given higher priority for matching, while trajectories with t>30 are abandoned. We considered that, with increasing *t*, the predictive reliability of trajectories gradually decreases. Hence, in the distance weighting, we linearly decrease the weight of distance *a* from 0.8 to 0. Additionally, as *t* increases, the distance threshold for cell movement should also increase. Under the conditions of two-dimensional Brownian motion, the displacement of cells within a time interval Δt follows a normal distribution with 2DΔt. Setting the distance threshold to the reference distance of Brownian motion is one of the main improvements of our algorithm. We calculate the diffusion coefficient for the current time based on the statistics of all cells in the previous three frames and set the distance threshold to $\sqrt{2D\Delta t}$. As show in Figure 6b, we can see that, after employing Distance Cascade Matching, we are able to effectively track fast-moving targets.

#### 3.2.2. Overall Suppression

When detecting small targets, especially in densely populated scenarios, we encounter false-positive detections, as illustrated in Figure 3c. The IoU value of this erroneous detection box, when compared individually with other correctly detected boxes, is relatively low. However, the sum of the IoU values of this erroneous detection box with the two other correct boxes is quite large. This observation has inspired us to implement a method called Overall Suppression in our tracking process.

Considering the nature of our tracking targets, NKCs neither suddenly appear nor disappear. When a highly overlapping detection box suddenly appears near an established trajectory, it is likely a false positive. Thus, in our tracking algorithm process, after target matching, we obtain established trajectories. We do not immediately designate unmatched detection boxes as “potential new trajectories”. Instead, we calculate the IoU with all successfully matched detection boxes, summing up the total IoU values. Detection boxes exceeding a threshold are directly removed. Only after this round of deletion can they be set as “potential new trajectories”. As shown in Figure 6e, this step effectively reduces erroneous tracking trajectories.

#### 3.2.3. Re-Search

As shown in Figure 3c, when cells are on the surface of cancer cells, we cannot predict their positions based solely on the current frame; instead, contextual information is required for inference. Therefore, we adopt a Re-Search strategy, inspired by the mechanism of human visual tracking. When tracking blurry small targets in the field of view, we do not globally search for the targets; instead, we scan the vicinity of the target’s location to find objects that significantly contrast with the background.

**Figure 7:**
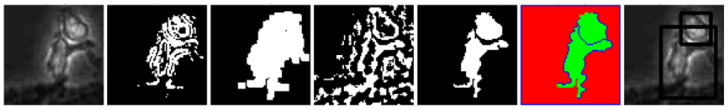
This image shows how we search for prominent objects near the target. From left to right, the images represent the original cell image, the cell boundary obtained through Sobel segmentation, the image mask formed after dilation, the boundary obtained through the adaptive method, the overlap between the mask and the boundary, the segmented image using the watershed algorithm, and the final drawn bounding box.

The implementation steps on our computer are illustrated in Figure 7. Firstly, we extract an 80 × 80 region near the area where the target was lost (approximately three times the size of the target). We then apply the Sobel operator for initial edge detection. Subsequently, a dilation algorithm is used to create an initial mask, effectively separating the foreground from the background. In cases where the target may be partially connected, further segmentation is required. Therefore, we employ an adaptive threshold segmentation method to achieve more precise boundaries. We calculate the intersection of edges and masks, and then utilize the watershed algorithm for the final segmentation of the image.

#### 3.2.4. Framework of Tracking Network

**Figure 8:**
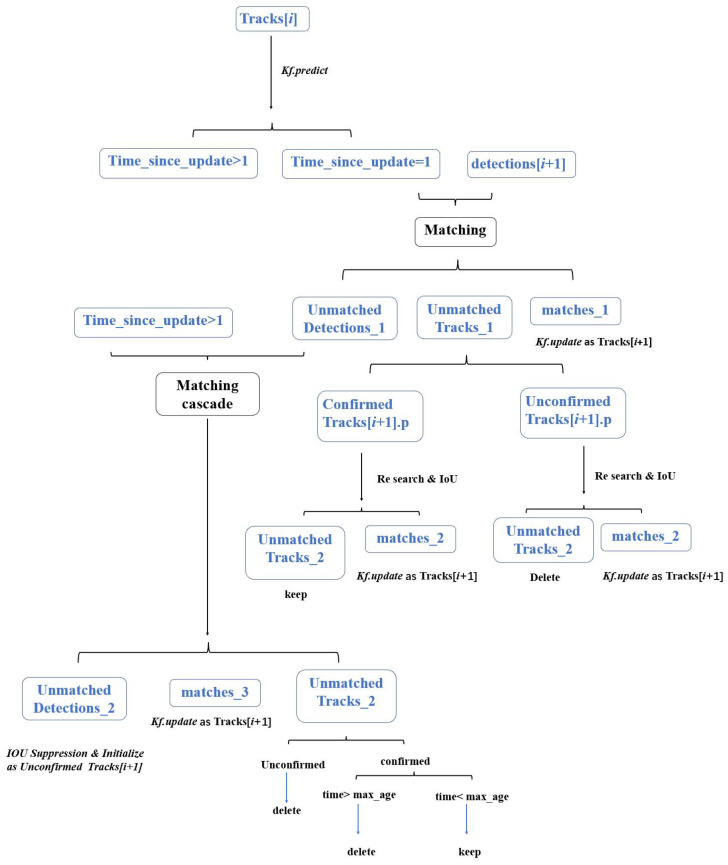
This figure illustrates how we perform the tracking process. “Tracks[i]” in the figure refers to the collection of target trajectory information up to the ith frame, including the position, velocity, and “time_since_update” information for each target. As the number of frames increases, we need to continuously update the motion status of each target. The “time_since_update” value reflects how long it has been since a target’s motion status was last updated, which can be understood as the time a target trajectory has been lost. “Detections[i + 1]” refers to the collection of targets detected in the (i + 1)th frame. Our algorithm process involves matching “Detections[i + 1]” with the historical trajectories “Tracks[i]” to update the targets’ status data. “Confirmed tracks” in the figure refer to the trajectories successfully tracked for more than three consecutive frames. Even if there are brief unsuccessful matches along the way, we retain the status of these trajectories. For “unconfirmed tracks”, if they lose their matches midway, we directly delete their trajectory information.

The entire tracking process combines the aforementioned methods, and the overall program framework is illustrated in Figure 8, explaining how we match the detection of frame I + 1 with the existing tracks up to frame I to update track information. We first classify the tracks in frame I into two categories based on the “time_since_update” value. A “time_since_update=1” indicates that the track has just been updated in frame I, meaning that it is present in this frame (as opposed to being lost from view). We prioritize matching these tracks. If this matching fails, we Re-Search near the track to locate significant targets and update successfully matched tracks, then compute the Intersection over Union (IoU) for Overall Suppression. Next, for tracks with “time_since_update>1”, we match them with unmatched detections through a distance-based cascading process. At this stage, successfully matched tracks will have their status updated. However, since these tracks do not have a corresponding detection in the frame I and retain only the previous track record, we do not Re-Search for them. At the same time, for tracks with continuous records over three or more frames, we mark them as confirmed tracks. Even if they are temporarily lost from view, their track information will be retained.

The corresponding pseudocode is provided in Appendix A. We evaluated the tracking performance of the new algorithm incorporating these three improvements. As shown in Table 1, its IDF1 score increased from 66.6% with ByteTrack to 81.5%. This indicates a significant improvement in the continuity and stability of the tracking algorithm. Additionally, the inclusion of the Re-Search and Overall Suppression mechanisms allows us to reduce detection errors to some extent, resulting in a slight increase in MOTA from 79.0% to 83.6%.

## 4. Results

### 4.1. Kinematic Differences between P-NKCs and NP-NKCs

**Figure 9:**
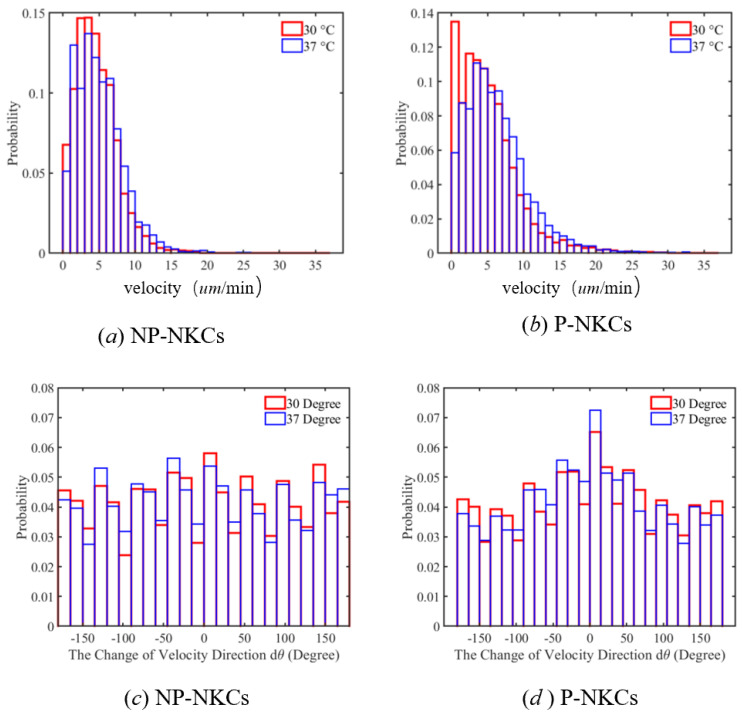
(**a**) The velocity distribution of NP-NKCs at different temperatures, showing a slight decrease in the proportion of cells in the low-speed region as the temperature rises. (**b**) The velocity distribution of P-NKCs at different temperatures, with a noticeable decrease in the proportion of cells in the low-speed region as the temperature increases. (**c**) The velocity deviation angle of NP-NKCs at different temperatures, where only the average of the deviation angles between consecutive frames is recorded. As the temperature rises, the distribution of deviation angles becomes more uniform. (**d**) The velocity deviation angle of P-NKCs at different temperatures, where, contrary to NP-NKCs, the deviation angles tend to concentrate more around 0° as the temperature increases.

**Figure 10:**
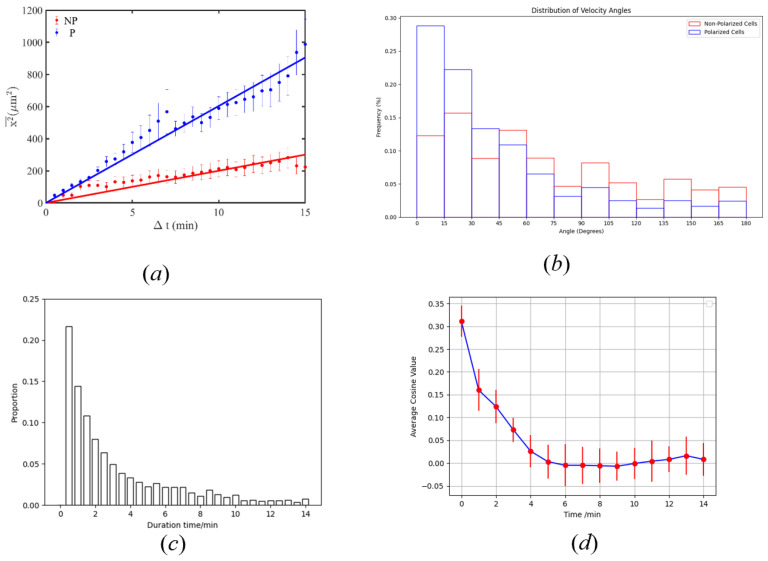
(**a**) presents the diffusion of P-NKCs and NP-NKCs, demonstrating that p-cells have a higher diffusion coefficient. (**b**) displays the frequency distribution of changes in the angle of velocity for the cells, indicating that P-NKCs are more likely to maintain their original direction of movement. (**c**) depicts the duration for which p-cells maintain their direction of velocity. (**d**) is the relationship between the angle of deviation in velocity direction between two instances and the current state with the time difference between the two velocities.

Our initial analysis focused on the motility characteristics of P-NKCs and NP-NKCs in an environment devoid of cancer cells. By examining the velocity distribution of natural killer cells, shown in Figure 9, we noted that P-NKCs demonstrated enhanced motility, with a significant presence in the high-speed region. This higher activity level of P-NKCs was also reflected in the mean square displacement (MSD, ${\bar{\chi }}^{2}$) versus time plot, as illustrated in Figure 10a, where we performed regression analysis on both cell types. Both of them exhibited a highly linear correlation, with correlation coefficients ($R^{2}$) of 0.98 for P-NKCs and 0.97 for NP-NKCs. We observed that the diffusion coefficient *D* of P-NKCs (59.62 μm^2^/s) was significantly higher than that of NP-NKCs (19.1 μm^2^/s).

Further investigation into the directional consistency of these cells’ movement revealed intriguing patterns in the velocity deviation angles, depicted in Figure 10b. It is observable that P-NKCs have a higher probability of maintaining their original direction of motion, whereas the velocity deviation distribution of NP-NKCs is more uniform. This result seems to indicate that the movement of NP-NKCs is nearly random, while the movement of P-NKCs is non-random. However, further analysis of the data suggests otherwise.

We measured the average angle between the polarization direction and velocity direction for these morphologically simple P-NKCs cells, resulting in a mean angle of 24° with a standard deviation of 31°. Subsequently, we measured the distribution of the duration for which polarized cells maintain their velocity. Here, we consider the direction of velocity to be consistent between two frames if the deviation is within 30°; deviations beyond 30° are considered a change in velocity direction. The distribution results, as shown in Figure 10c, indicate that the direction of velocity of polarized cells is maintained for an average duration of 4 min.

Lastly, by assessing the average cosine of the velocity deviation angle over time, illustrated in Figure 10d, we observed a notable decay in directional consistency over time. Combining these observations, we believe that polarized cells tend to move along the polarization direction, and, in the absence of external stimuli, the polarized state of natural killer cells is maintained for a certain period. This maintenance of polarization explains why P-NKCs exhibit a consistent direction of velocity over time. However, over longer periods, the direction of velocity of polarized cells remains random and disorderly, which is also evident from the MSD–time regression curves.

Combining all the data mentioned above, we conclude that, when there is no external stimulus, the movement of natural killer cells follows a random Brownian motion, governed by the Langevin equation:

$$\frac{d}{dt}u\left(t\right)=-\gamma u\left(t\right)+\frac{1}{m}R\left(t\right)$$

where *u* is the cell velocity, m is the mass, γ is the viscous drag coefficient, and R(t) is the random force following a Gaussian distribution. For P-NKCs, due to changes in their morphology, they have a smaller γ in their polarized direction, causing them to tend to move along the polarization direction during motion, with a higher velocity compared to NP-NKCs [32]. However, the polarization direction of P-NKCs is not fixed but randomly changes. Over a longer period, both follow the solution of the Langevin equation: $\chi^{2}=2D\Delta t$, where Δt represents the time interval and *D* is the diffusion coefficient. Additionally, P-NKCs have a higher *D*.

### 4.2. Impact of Temperature

In the experiment, we compared the motion of immune cells at 30 degrees Celsius and 37 degrees Celsius, both in environments without cancer cells. At these temperatures, cells were able to maintain basic activity. First, we measured the velocity distribution of cells at different temperatures, and the results are shown in Figure 9. We observed that, as the temperature increased, both P-NKCs and NP-NKCs exhibited an increase in velocity. However, compared to NP-NKCs, P-NKCs showed a more significant increase in velocity. It can be seen that the distribution of cells in the low-speed region decreased significantly.

Interestingly, as the temperature increases, the distribution of velocity deviation angles for NP-NKCs becomes more random, aligning with classical thermodynamic laws. However, the distribution of velocity deviation angles for P-NKCs becomes more concentrated around 0, indicating a tendency to maintain their original direction of velocity. We believe this is what distinguishes polarized cells from non-living molecular phenomena. Due to their inclination to move along the polarization direction, the activity and persistence of this polarization are enhanced with rising temperatures, increasing the probability of maintaining their direction of motion.

### 4.3. Impact of Tumor Cell

**Figure 11:**
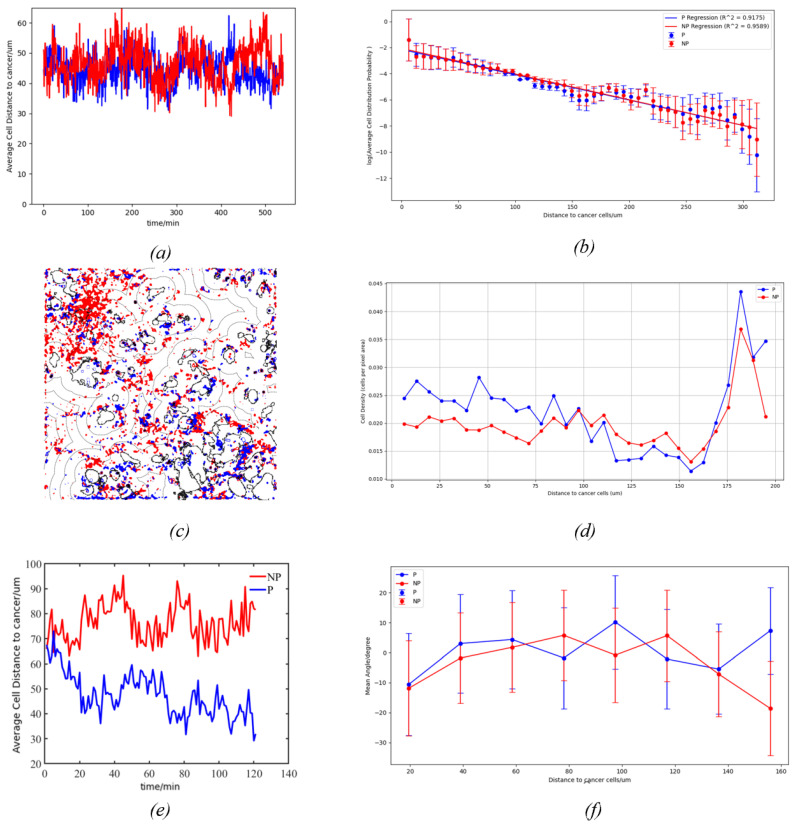
(**a**) The relationship between the average distance of NKCs and time, where blue corresponds to P-NKCs and red corresponds to NP-NKCs. The same color scheme applies to the other figures. (**b**) The relationship between the average number distribution of NKCs per frame and distance, with the standard deviation calculated across different frames. The y-axis is logarithmically scaled and regressed. (**c**) The total number distribution of NKCs in the cancer cell environment, where the dashed black lines in the figure represent equidistant lines to the surface of the cancer cells. Note that this figure does not represent the average number distribution of cells in a single frame but is formed by overlaying the cell numbers from all time points. (**d**) The relationship between the average density distribution of NKCs per frame and distance. (**e**) This figure shows the change in average distance over time for two types of cells. It can be observed that P-NKCs reduce their distance to cancer cells more quickly as time progresses. (**f**) The relationship between the velocity deviation angle of NKCs and their distance to cancer cells.

We first measured the average distance of NKCs to the cancer cell surface within 2 h after introduction into the cancer cell environment, with a total of 241 frames captured. The results, depicted in Figure 11e, show that the average distance of NP-NKCs to the nearest cancer cell surface oscillated around 75 μm over time. In contrast, the average distance of P-NKCs to the nearest cancer cell surface gradually decreased over time, indicating a tendency to approach the cancer cells. However, when observing the data after sufficient mixing of NKCs with cancer cells, the tracked statistical results, as shown in Figure 11a, indicate that the average distances of both NP-NKCs and P-NKCs oscillated within the range of 30 to 60 μm over time, with no significant difference observed in their distributions. As there was no longer a trend of increasing or decreasing average distances, we conclude that the motion of NKCs under cancer cells reached a dynamic equilibrium state at this point.

Subsequently, we further analyzed the data of NKCs in the equilibrium state. We calculated the total number distribution of natural killer (NK) cells in the cancer cell environment, as depicted in Figure 11c. It is noticeable that NKCs are concentrated in areas that are densely populated with cancer cells. We quantified this by plotting the relationship between the distribution ratio of NKCs and their distance, as shown in Figure 11b. We can see that the log-transformed number distribution of NKCs shows a significant linear correlation with distance. However, we further considered that the areas enclosed by different distance intervals to cancer cells have different areas, necessitating the calculation of cell density distribution rather than just the number distribution, with the results shown in Figure 11d. We can see that the density distribution exhibits an irregular smooth distribution, and the effect of linear fitting after log transformation is not good.

We posit that this phenomenon occurs because, in the cancer cell environment, cancer cells can secrete certain chemicals. In a state of equilibrium, these chemicals have a distribution V(x), which can attract NKCs. This gradient distribution can generate a directed driving force on natural killer cells:

K(x)=−∇V(r)

In this way, the Langevin equation can be writen as

$$\frac{d}{dt}u\left(t\right)=-\gamma u\left(t\right)+\frac{1}{m}\left[R\left(t\right)+K\left(x\right)\right]$$

We can deduce that, in the equilibrium state, the distribution of natural killer cells should follow

$$n\left(r\right)=n_{0}e^{-\frac{V(r)}{k_{B}T}}$$

The distribution frequency of NKCs conforms to our Boltzmann distribution hypothesis, while the distribution density of NKCs has no obvious pattern. We believe that this is because, in each individual frame, the number of cells compared to the area they occupy is still very sparse. When the number of cells is divided by the vast area, the impact of the number of cells is flattened, and what absolutely affects the value of cell density is instead the size of the area (this can also be seen from the sudden increase in the density curve later on).

Finally, we investigated whether cancer cells exert a deflecting effect on NKCs by measuring the average deviation angle of NKCs at varying distances from the cancer cell surface. Negative values indicate NKCs deviating towards cancer cells, while positive values signify moving away, as illustrated in Figure 11d. There is a clear attraction at close distances to cancer cells, which rapidly diminishes as the distance increases.

## 5. Conclusions

In this study, we focused on the different morphologies’, temperatures’, and cancer cell environments’ impact on natural killer cell motion. Leveraging machine learning techniques, we process time-lapse microscopy images of NKCs provided by Professor Jue Shi from the Hong Kong Baptist University to efficiently acquire corresponding immunological data for analysis. In our program design, we divide the cell tracking process into single-frame target detection and data association between multiple frames, enabling both cell morphology analysis and cell tracking. For single-frame target detection, we employ YOLOv5 to process NKCs images, achieving over 92% target detection accuracy. Addressing the challenges of small NKC targets, blurry morphologies, and complex backgrounds, we improve upon existing data association methods like DeepSORT and ByteTrack by introducing Distance Cascade Matching, Overall Suppression, and Re-Search methods for multi-frame data association. Ultimately, our algorithm achieved a MOTA (Multiple Object Tracking Accuracy) of 83.6% and an IDF1 (ID F1 score) of 81.5%, representing significant improvements compared to previous algorithms, which enable precise tracking of NKCs.

We observed that P-NKCs exhibited stronger motility and could maintain their polarization direction for a certain period in the absence of cancer cell stimulation. A moderate increase in temperature enhanced the motility of natural killer cells, especially under polarizing conditions. In the presence of cancer cells, at equilibrium, the number of natural killer cells shows an exponential decay distribution with distance, and cancer cells exert an attractive effect on the motion of natural killer cells. However, this influence is limited, and it becomes less pronounced beyond a distance of 30 micrometers.

This article has some limitations. While we observed a correlation between the motion direction of simple polarized cells and their polarization direction, and this polarization direction could be maintained to some extent, this explains well why, with the increase in temperature, the velocity consistency of polarized cells is actually higher. However, we were unable to analyze the morphology of more complexly polarized cells. When there are multiple polarization directions, this may indicate that they are simultaneously stimulated by chemotactic factors from multiple directions. At this point, the choice of polarization direction for polarized cells is worth investigating, and this will be the direction of our further research in the future.

## Figures and Tables

**Table 1:** The performance of DeepSORT, ByteTrack, and our new tracking algorithm on NKCs’ data and MOT17.

	MOT17			NKCs’ Data		
**Tracker**	**MOTA**	**IDF1**	**IDs**	**MOTA**	**IDF1**	**IDs**
DeepSORT	75.4%	77.0%	238	68.7%	42.7%	1948
ByteTrack	76.3%	80.5%	216	79.0%	66.6%	411
NEW TRACKING	/	/	/	83.6%	81.5%	149

## Data Availability

The relevant experimental data are available from a repository, namely, NKCs DAT, and can be accessed through the following link: https://github.com/lx9days/trackingCode (accessed on 1 May 2024).

## Appendix A

Here is the pseudocode that showcases our overall tracking process.

**Algorithm A1**: Pseudo-code of NKCs tracking

## References

[1] Li Y., Wu X., Sheng C., Liu H., Liu H., Tang Y., Liu C., Ding Q., Xie B., Xiao X. (2024). IGSF8 is an innate immune checkpoint and cancer immunotherapy target. Cell.

[2] Shin E., Yoon S.-R., Noh J.-Y. (2023). Understanding NK Cell Biology for Harnessing NK Cell Therapies: Targeting Cancer and Beyond. Front. Immunol..

[3] Wolf N.K., Kissiov D.U., Raulet D.H. (2023). Roles of natural killer cells in immunity to cancer, and applications to immunotherapy. Nat. Rev. Immunol..

[4] Mahoney K.M., Rennert P.D., Freeman G.J. (2015). Combination cancer immunotherapy and new immunomodulatory targets. Nat. Rev. Drug Discov..

[5] Zheng X., Hou Z., Qian Y., Zhang Y., Cui Q., Wang X., Shen Y., Liu Z., Zhou Y., Fu B. (2023). Tumors evade immune cytotoxicity by altering the surface topology of NK cells. Nat. Immunol..

[6] Watzl C., Sternberg-Simon M., Urlaub D., Mehr R. (2012). Understanding natural killer cell regulation by mathematical approaches. Front. Immunol..

[7] Hancioglu B., Swigon D., Clermont G. (2007). A dynamical model of human immune response to influenza A virus infection. J. Theor. Biol..

[8] Wodarz D., Sierro S., Klenerman P. (2007). Dynamics of killer T cell inflation in viral infections. J. R. Soc. Interface.

[9] Elemans M., Thiébaut R., Kaur A., Asquith B. (2011). Quantification of the relative importance of CTL, B cell, NK cell, and target cell limitation in the control of primary SIV-infection. PLoS Comput. Biol..

[10] Jiang Y., Hou X., Zhao X., Jing J., Sun L. (2023). Tracking adoptive natural killer cells via ultrasound imaging assisted with nanobubbles. Acta Biomater..

[11] Zhang Y., Wang C., Wang X., Zeng W., Liu W. (2020). A Simple Baseline for Multi-Object Tracking. arXiv.

[12] Girshick R., Donahue J., Darrell T., Malik J. Rich Feature Hierarchies for Accurate Object Detection and Semantic Segmentation. Proceedings of the 2014 IEEE Conference on Computer Vision and Pattern Recognition.

[13] Redmon J., Farhadi A. (2018). YOLOv3: An Incremental Improvement. arXiv.

[14] Lin T.Y., Goyal P., Girshick R., He K., Dollár P. (2020). Focal Loss for Dense Object Detection. IEEE Trans. Pattern Anal. Mach. Intell..

[15] Zhou X., Li S., Wang X., Zhou Q. TPH-YOLOv5: Improved YOLOv5 Based on Transformer Prediction Head for Object Detection on Drone-captured Scenarios. Proceedings of the 2021 IEEE/CVF International Conference on Computer Vision Workshops (ICCVW).

[16] Mirzaei B., Nezamabadi-Pour H., Raoof A., Derakhshani R. (2023). Small Object Detection and Tracking: A Comprehensive Review. Sensors.

[17] Marvasti-Zadeh S.M., Cheng L., Ghanei-Yakhdan H., Kasaei S. (2021). Deep learning for visual tracking: A comprehensive survey. IEEE Trans. Intell. Transp. Syst..

[18] Guo S., Wang S., Yang Z., Wang L., Zhang H., Guo P., Gao Y., Guo J. (2022). A review of deep learning-based visual multi-object tracking algorithms for autonomous driving. Appl. Sci..

[19] Chunlei L., Wenrui D., Jinyu Y., Vittorio M., Baochang Z., Jungong H., Guodong G. (2020). Aggregation Signature for Small Object Tracking. IEEE Trans. Image Process..

[20] Zhu Y., Huang B., Shi J. (2016). Fas ligand and lytic granule differentially control cytotoxic dynamics of natural killer cell against cancer target. Oncotarget.

[21] Zhu Y., Xie J., Shi J. (2021). Rac1/ROCK-driven membrane dynamics promote natural killer cell cytotoxicity via granzyme-induced necroptosis. BMC Biol..

[22] He K., Zhang X., Ren S., Sun J. Deep Residual Learning for Image Recognition. Proceedings of the 2016 IEEE Conference on Computer Vision and Pattern Recognition (CVPR).

[23] Liu W., Anguelov D., Erhan D., Szegedy C., Reed S., Fu C.-Y., Berg A.C. SSD: Single Shot MultiBox Detector. Proceedings of the Computer Vision–ECCV 2016: 14th European Conference.

[24] Li T., Zhang Z., Pei L., Gan Y. (2022). HashFormer: Vision Transformer Based Deep Hashing for Image Retrieval. IEEE Signal Process. Lett..

[25] Xu J., Zhou W., Chen Z. (2021). Blind Omnidirectional Image Quality Assessment With Viewport Oriented Graph Convolutional Networks. IEEE Trans. Circuits Syst. Video Technol..

[26] Redmon J., Divvala S., Girshick R., Farhadi A. You only look once: Unified, real-time object detection. Proceedings of the IEEE Conference on Computer Vision and Pattern Recognition.

[27] Maji D., Nagori S., Mathew M., Poddar D. YOLO-Pose: Enhancing YOLO for Multi Person Pose Estimation Using Object Keypoint Similarity Loss. Proceedings of the IEEE/CVF Conference on Computer Vision and Pattern Recognition.

[28] Bernardin K., Stiefelhagen R. (2008). Evaluating multiple object tracking performance: The CLEAR MOT metrics. EURASIP J. Image Video Process..

[29] Ristani E., Solera F., Zou R., Cucchiara R., Tomasi C. (2016). Performance measures and a data set for multi-target, multi-camera tracking. Proceedings of the European Conference on Computer Vision.

[30] Wojke N., Bewley A., Paulus D. Simple Online and Realtime Tracking with a Deep Association Metric. Proceedings of the 2017 IEEE International Conference on Image Processing (ICIP).

[31] Zhang Y., Sun P., Jiang Y., Yu D., Wang F., Yuan Z., Liu P., Liu W., Wang X. (2022). ByteTrack: Multi-Object Tracking by Associating Every Detection Box. Proceedings of the European Conference on Computer Vision.

[32] Jilkine A., Edelstein-Keshet L. (2011). A Comparison of Mathematical Models for Polarization of Single Eukaryotic Cells in Response to Guided Cues. PLoS Comput. Biol..

